# Compound optimization through data set-dependent chemical transformations

**DOI:** 10.1186/1758-2946-6-S1-P5

**Published:** 2014-03-11

**Authors:** Antonio de la Vega de León, Jürgen Bajorath

**Affiliations:** 1 Department of Life Science Informatics, B-IT, LIMES Program Unit Chemical Biology and Medicinal Chemistry, Rheinische Friedrich-Wilhelms-Universität, Dahlmannstrasse 2, D-53113 Bonn, Germany

## 

Matched molecular pairs (MMPs) have previously been used to extract chemical transformations and study their effect on molecular properties such as activity [1,2]. Chemical transformations have been used to direct compound optimization efforts towards defined activity profiles [3]. Here we introduce a methodology to assess effects of chemical transformations based on MMPs of compounds active against specific targets. The effects of selected chemical transformations on drug design-relevant molecular properties were analyzed. For different data sets, transformations that were frequently found and induced favorable property changes were identified. These transformations were then iteratively applied to modify active compounds and move them into favorable regions of ADME-relevant property space. Activity of newly designed compounds was tracked using nearest-neighbor searches in ChEMBL. The results of our study indicate that activity-conservative data-set dependent transformation can aid in the design of new active compounds with favorable ADME characteristics [4].
